# Design and analysis of DNA strand displacement devices using probabilistic model checking

**DOI:** 10.1098/rsif.2011.0800

**Published:** 2012-01-04

**Authors:** Matthew R. Lakin, David Parker, Luca Cardelli, Marta Kwiatkowska, Andrew Phillips

**Affiliations:** 1 Microsoft Research, 7 JJ Thomson Avenue, Cambridge CB3 0FB, UK; 2 Department of Computer Science, University of Oxford, Wolfson Building, Parks Road, Oxford OX1 3QD, UK; 3 Department of Computer Science, University of New Mexico, Albuquerque, NM 87131, USA

**Keywords:** DNA computing, formal verification, probabilistic model checking, DNA strand displacement

## Abstract

Designing correct, robust DNA devices is difficult because of the many possibilities for unwanted interference between molecules in the system. DNA strand displacement has been proposed as a design paradigm for DNA devices, and the DNA strand displacement (DSD) programming language has been developed as a means of formally programming and analysing these devices to check for unwanted interference. We demonstrate, for the first time, the use of probabilistic verification techniques to analyse the correctness, reliability and performance of DNA devices during the design phase. We use the probabilistic model checker prism, in combination with the DSD language, to design and debug DNA strand displacement components and to investigate their kinetics. We show how our techniques can be used to identify design flaws and to evaluate the merits of contrasting design decisions, even on devices comprising relatively few inputs. We then demonstrate the use of these components to construct a DNA strand displacement device for approximate majority voting. Finally, we discuss some of the challenges and possible directions for applying these methods to more complex designs.

## 1. Introduction

Molecular computing is a relatively new field that aims to construct information-processing devices at the molecular level. In particular, molecular devices constructed using DNA show promise for a wide range of important application areas, including biosensing, biomimetic molecular manufacture and drug delivery. However, designing correct and robust DNA devices is a major challenge. This results, in part, from the possibility of unwanted interference between molecules in the system. The DNA strand displacement (DSD) [1,2] has been developed to facilitate the design, simulation and analysis of DNA strand displacement devices.

In this paper, we propose the use of *formal verification* techniques to check the correctness of, and identify faulty behaviour in, DNA device designs. We focus on *model checking*, a fully automated approach to verification based on the exhaustive exploration of a finite-state model. We also employ *probabilistic model checking*, which generalizes these techniques to the analysis of probabilistic models of systems that exhibit stochastic behaviour, for example, owing to the possibility of failures or uncertainty regarding timing. Conventional (non-probabilistic) model-checking techniques can be used to check correctness properties such as ‘molecules 1 and 2 are never simultaneously bound to molecule 3’, whereas probabilistic model checking allows verification of *quantitative* guarantees such as ‘the probability of a strand displacement device failing to complete within 20 min is at most 10^−6^’. Furthermore, probabilistic model checking can be used to evaluate many other quantitative properties, such as performance: ‘what is the expected time for an input signal to be transduced to an output signal by a strand displacement circuit?’. More generally, probabilistic model checking has already been successfully applied to the analysis of systems from a wide range of application areas, from communication protocols such as Bluetooth [3] to pin-cracking attacks for cash machines [4]. In particular, it has also been used in the domain of systems biology to analyse, for example, cell signalling pathways [5,6]. In this paper, we use the probabilistic model-checking tool prism  [7].

The remainder of the paper is structured as follows. In §2, we present DNA strand displacement and the DSD programming language, and introduce probabilistic model checking and the prism model checker. In §3, we present the results of applying probabilistic model checking to DNA strand displacement gates and systems, including a DNA strand displacement device for approximate majority voting. Additional details of the methods are presented in §4, followed by a discussion of future work in §5.

## 2. Background

### 2.1. DNA strand displacement

DNA strand displacement [8] is a mechanism for performing computation with DNA molecules. Once initial species of DNA are mixed together, strand displacement systems proceed autonomously [9] as increases in entropy (from releasing strands) and enthalpy (from forming additional base pairs) drive the system forward [10]. These increases typically result from the conversion of active gate structures into unreactive waste. Furthermore, because DNA strand displacement relies solely on hybridization between complementary nucleotide sequences to perform computational steps, these systems require no additional enzymes or transcription machinery, which in turn allows experiments to be run using simple laboratory equipment.

In most strand displacement schemes, populations of single strands of DNA are interpreted as signals, whereas double-stranded DNA complexes act as gates, mediating changes in the signal populations. Within the system, the computational mechanism is *toehold-mediated branch migration and strand displacement* [11]. At the periphery of the system, signal populations may be connected to fluorophores for human-readable output, or regulated by custom-designed aptamer molecules that interface to the biological environment. The latter example highlights a key strength of DNA-based computational devices: the ability to interface directly with biological systems [12,13].

**Figure 1.**
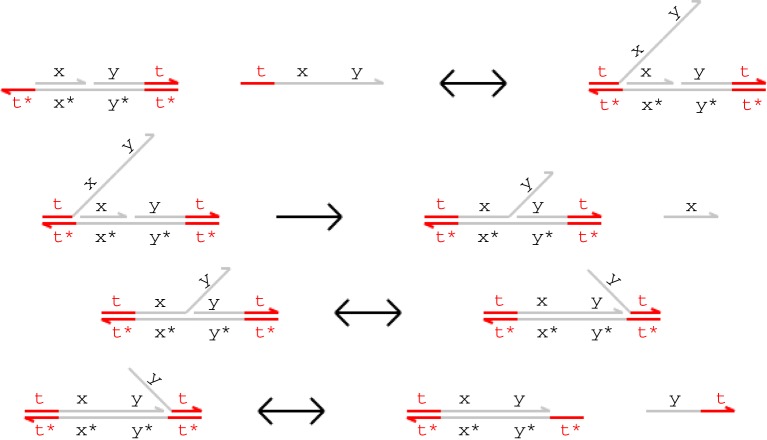
Toehold-mediated DNA branch migration and strand displacement. (Online version in colour.)

Figure 1 presents example branch migration and strand displacement reactions. Each letter in the figure represents a distinct domain (a sequence of nucleotides) and the asterisk operator (*) denotes the Watson–Crick (C–G, T–A) complement of a given domain. Short domains (represented in colour) are known as *toeholds*, while long domains (in grey) are often referred to as *recognition domains*. We assume that toeholds are sufficiently short (4–10 nucleotides) that they hybridize reversibly with their complements, whereas recognition domains are sufficiently long (greater than 20 nucleotides) to hybridize irreversibly [11]. Each single strand is oriented from the 5′ (left) end to the 3′ (right) end, and each double-stranded complex consists of hybridized single strands with opposite orientations. We assume that the underlying nucleotide sequences have been chosen such that distinct domains do not interact at all.

In the first reaction from figure 1, an incoming strand binds to a gate because the ‘t’ toehold domain in the strand hybridizes with its exposed complement in the gate, producing the intermediate complex on the right-hand side. Because the incoming strand is only held on by a toehold, this reaction can be reversed, causing the single strand to float away into the solution. In the second reaction, the ‘x’ domain in the overhanging strand matches the next domain along the double-stranded backbone, which means that the branching point between the overhanging strand and the backbone can move back and forth in a random walk called a *branch migration*. Eventually, the random walk may completely detach the short ‘x’ strand from the gate in a *strand displacement*. This reaction is considered irreversible because the invading strand is now attached to the gate by a long domain as well as a toehold. Note that if the recognition domain on the strand did not match the next domain along the gate, then branch migration could not proceed, and the incoming strand would eventually unbind. We call such binding reactions *unproductive*. The third reaction is another branch migration, though in this case no strand is displaced because even after the ‘y’ domain has been displaced, the rightmost strand is still attached by a toehold. The fourth reaction is a (reversible) unbinding reaction in which the rightmost strand spontaneously unbinds because of the low binding strength of the toehold.

Binding, migration and unbinding reactions such as those illustrated in figure 1 allow signal populations to be dynamically modified over time, and irreversible strand displacement reactions such as the second reaction from figure 1 provide a thermodynamic bias towards producing output. Combining these different kinds of reactions allows us to construct cascades of gates in which the output strands from one gate serve as the input strands for another. This technique has enabled the construction of large, complex logic circuits based on DNA strand displacement [14].

In this paper, we restrict our attention to a class of gates closely related to Cardelli's two-domain gate scheme [15]. Two-domain gates are a restricted class of systems where every strand consists of two domains (a toehold and a long recognition domain) and gates have no structures hanging off the main double-stranded backbone of the complex. The initial and final gates shown in figure 1 have this property, although the intermediate steps do involve transient overhanging structures during branch migration and strand displacement steps. These gate structures can be thought of as one continuous strand hybridized with a complementary strand that has breaks at certain points. Such restricted gate structures could be assembled by synthesizing double-stranded DNA and inserting the breaks using either restriction enzymes or site-specific photocleavage to split the backbone of the DNA strand at the appropriate point. This technique should allow gates to be constructed with a higher yield than would be obtained with the usual technique of annealing single strands, which has a higher probability of producing unwanted secondary structures. In this paper, we use a variant of two-domain gates that relaxes the restrictions on single-stranded DNA molecules but that retains the simplified gate structure with all its practical benefits to the experimentalist.

### 2.2. The DNA strand displacement programming language

The DSD programming language [2] provides a textual syntax for expressing the structure of DNA species such as those portrayed graphically in figure 1. The semantics of the DSD language defines a formal translation of a collection of DNA species into a system of chemical reactions that captures the possible interactions between the species. The language includes syntactic and graphical abbreviations that allow us to represent a particular class of DNA molecules in a concise manner. The class of molecules in question is those without secondary structure—that is, only single-stranded DNA sequences may hang off the main double-stranded backbone of the molecule. This rules out tree-like or pseudo-knotted structures, which greatly simplifies the definition of the semantics while still allowing a wide variety of systems to be designed.

The textual syntax of the DSD programming language and the corresponding graphical representation are presented in table 1. The syntax is defined in terms of sequences S, L, R, strands A, gates G and systems D. A sequence S comprises one or more domains, which can be long domains N or short domains N^∧^. DNA species can be single or double stranded. A single upper strand 〈S〉 denotes a sequence S oriented from left to right on the page, while a single lower strand {S} denotes a sequence S oriented from right to left on the page. A double strand [S] denotes an upper strand 〈S〉 bound to the complementary lower strand {S*}. A gate G is composed of double-stranded segments of the form {L'}〈L〉[S]〈R〉{R'}, which represents an upper strand 〈L S R〉 bound to a lower strand {L' S* R'} along the double-stranded region [S]. The sequences L, R, L′ and R' can potentially be empty, in which case we simply omit them. Gates are built up by concatenating gate segments G1 and G2 along a common lower strand, written G1:G2, or along a common upper strand, written G1::G2. In the graphical representation, we omit the colons altogether and connect the strands.

**Table 1.** Syntax of the DNA strand displacement (DSD) language, in terms of strands A, gates G and systems D. Where present, the graphical representation below is equivalent to the program code above.

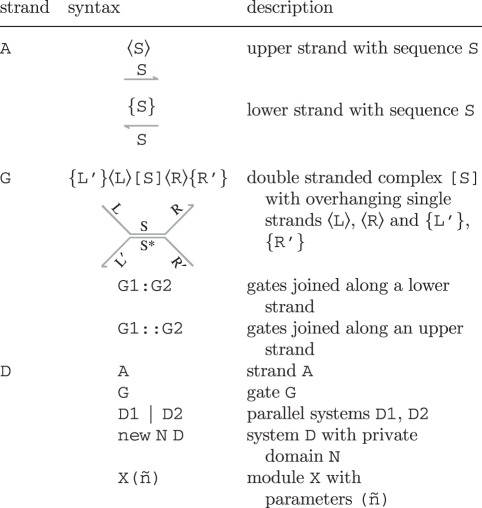

An individual DNA species can be an upper strand 〈S〉, a lower strand {S} or a gate G. We let D range over *systems* of such species. Multiple systems D1, D2 can be present in parallel, written as D1|D2. A domain N can also be restricted to molecules D, written new N D. This represents the assumption that the domain (or its complement) is not used by any other molecules outside D. We also allow module definitions of the form X(ñ)=D, where ñ are the module parameters and X(m̃) is an instance of the module D with parameters ñ replaced by m̃. We assume a fixed set of module definitions, which are declared at the start of the program. The definitions are assumed to be non-recursive, such that a module cannot invoke itself, either directly or indirectly via another module.

All of the models discussed in this paper were created using the Visual DSD tool.^1^ This is a web-based implementation of the DSD language that allows networks of strand displacement reactions to be designed, simulated and analysed. For the purposes of this work, we have developed additional functionality for visual DSD that allows the reaction network to be exported as a model that can be loaded into the prism probabilistic model checker for verification. Additional details of how reaction networks are computed in DSD are provided in §4.

### 2.3. Probabilistic model checking

*Model checking* is an automated formal verification technique, based on the exhaustive construction and analysis of a finite-state model of the system being verified. The model is usually a labelled state-transition system, in which each state represents a possible configuration of the system and each transition between states represents a possible evolution from one configuration to another. The desired correctness properties of the system are typically expressed in temporal logics, such as computation tree logic (CTL) or linear-time temporal logic. We omit here a precise description of these logics (see [16,17] for detailed coverage); instead, we give some typical CTL formulae below, along with their corresponding informal meanings:

— A [ G !(“access1” & “access2”): ‘processes 1 and 2 never simultaneously access a shared resource’;
— A [ F “end” ]: ‘the algorithm always eventually terminates’ and
— E [ !“fail” U “end” ]: ‘it is possible for the algorithm to terminate without any failures occurring’.

Once the desired correctness properties of the system have been formally expressed in this way, they can then be verified using a *model checker*. This performs an exhaustive analysis of the system model, for each property either concluding that it is satisfied or, if not, providing a counterexample illustrating why it is violated.

*Probabilistic model checking* is a generalization of model checking for the verification of systems that exhibit stochastic behaviour. In this case, the models that are constructed and analysed are augmented with quantitative information regarding the likelihood that transitions occur and the times at which they do so. In practice, these models are typically Markov chains or Markov decision processes. To model systems of reactions at a molecular level, the appropriate model is *continuous-time Markov chains* (CTMCs), in which transitions between states are assigned (positive, real-valued) rates. These values are interpreted as the rates of negative exponential distributions.

Properties of CTMCs are, like in non-probabilistic model checking, expressed in temporal logic, but are now quantitative in nature. For this, we use probabilistic temporal logics such as continuous stochastic logic (CSL) [18,19] and its extensions for reward-based properties [20]. For example, rather than verifying that ‘the protein always eventually degrades’, using CSL allows us to ask ‘what is the probability that the protein eventually degrades?’ or ‘what is the probability that the protein degrades within *t* hours?’ Reward-based properties include ‘what is the expected time that proteins are bound within the first *t* time units?’ and ‘what is the expected number of phosphorylations before relocation occurs?’ For further details on probabilistic model checking of CTMCs, see [19,20]. For a description of the application of these techniques to the study of biological systems, see Kwiatkowska *et al.*  [21]. All of the models discussed in this paper were analysed using prism [7], a probabilistic model-checking tool developed at the Universities of Birmingham and Oxford. Additional details of probabilistic model checking using prism are provided in §4.

## 3. Results

In this section, we present a series of case studies that demonstrate the application of probabilistic model-checking techniques to the design of DNA strand displacement systems. As mentioned previously, to do so we have extended the Visual DSD tool [2] with the capability to generate, from DSD designs, corresponding model descriptions that can be directly analysed by the prism probabilistic model checker [7]. We will study our modified two-domain gate designs from §2.1, and present analyses of various correctness, reliability and performance properties of the gates using prism. Finally, we will construct an approximate majority voting system using these components and show how additional approximation mechanisms can be adopted in order to verify this system.

### 3.1. Transducer gates: correctness

We begin by considering one of the simplest reaction gates: a transducer that takes an *X* signal as input and produces a *Y* signal as output. The gate can be thought of as implementing a unary chemical reaction *X* → *Y*. We will demonstrate the use of (non-probabilistic) model checking to debug strand displacement systems, by detecting a bug in a flawed design for a transducer gate.

**Figure 2.**
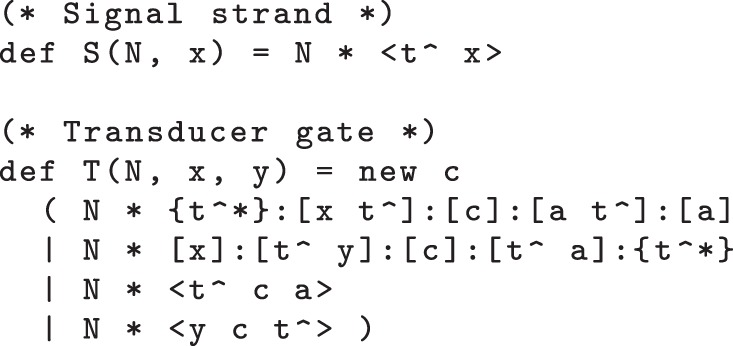
Initial transducer gate code, with additional definition for signal strands.

**Figure 3.**
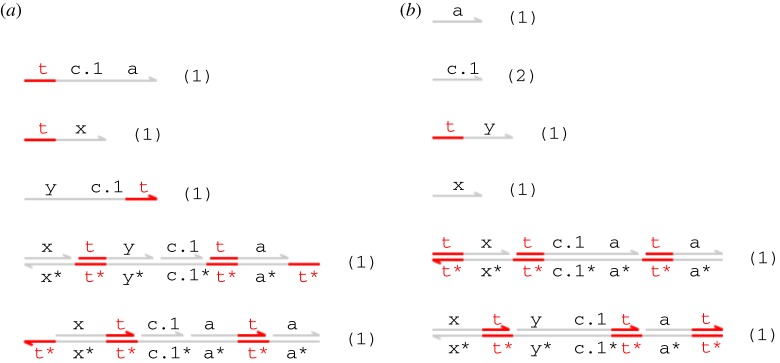
(*a*) Initial species and (*b*) expected final species for the transducer gate. (Online version in colour.)

Our initial transducer design is specified by the DSD code of figure 2. This also includes a definition of single-stranded signals, where the S(N,x) module denotes a population of *N* single-stranded signals carrying the *X* domain. We assume that the t^∧^ toehold is defined globally and shared by all gates and strands. The T(N,x,y) module represents *N* parallel copies of the transducer gate that implements the *X* → *Y* reaction. The body of the module definition consists of two populations of *N* complexes, and two populations of *N* strands. The species present in the initial state (when *N* = 1) are shown in figure 3*a*. The first gate accepts an input signal *X* and the second gate produces an output signal *Y*. The single strands are fuel species: the first ejects an intermediate strand from the input gate in an irreversible reaction that prevents input signal *X* from being rejected (thereby undoing the execution of the gate so far), whereas the second ejects the output signal *Y* from the output gate. This design is closely related to the two-domain design from [22], with the addition of a private ‘c’ domain that introduces an additional irreversible step into the execution of the gate. The effect of this modification is limited for the simple transducer gate but will become more apparent when we move on to more complicated gate designs. Note, however, that the definition of T(N,x,y) does not include a similar ‘new’ declaration for the ‘a’ domain. This has implications for crosstalk between gate populations, as we shall see below.

The expected final species for the transducer gate design (when *N* = 1) are shown in figure 3*b*. The intended effect is to convert an incoming 〈t^∧^ x〉 signal into 〈t^∧^ y〉, leaving only unreactive waste. We say that a strand or gate is *reactive* if it can react with some other species present in the system to cause a strand to be displaced, and *unreactive* otherwise. In this example, the unreactive species are those in which all toeholds occur in double-stranded segments and are thus sequestered.

Here, we will focus on verifying the correctness of two transducer gates in series. The first gate should turn a signal *X*_0_ into *X*_1_, and then the second should turn signal *X*_1_ into *X*_2_. Using the DSD code from figure 2, the input signal and pair of transducers are given by: S(1,x0) | T(1,x0,x1)| T(1,x1,x2).

To formalize a correctness property to be checked by prism, we first need to identify the states of the model in which the execution of the gates has completed successfully. This is performed with the following prism code, which is, in a generic form, designed to be applicable to various different designs:


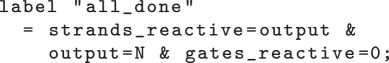


Here, strands_reactive and gates_reactive are pre-defined formulae (automatically generated by Visual DSD) that, when evaluated in a particular state of a model, return the number of reactive strands and reactive gates in that state, respectively. The variable output gives the number of output strands (in this example, 〈t^∧^ x2〉) and N is the number of parallel copies of the system. So, we say that the execution was successful when all copies of the gate have produced the required output and there are no reactive gates or strands (other than output strands) present.

Notice that, by definition, when the specification “all_done” given above is true; no further reactions can occur. Hence, such states of the model are *deadlock* states (those with no outgoing transitions to other states). We specify the *correctness* of the system design by stating that: (i) any possible deadlock state that can be reached must satisfy “all_done” and (ii) there is at least one path through the system that reaches a state satisfying “all_done”. These two properties can be represented by formulae in the (non-probabilistic) temporal logic CTL, which can be verified by prism:


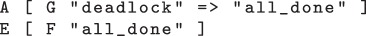


**Figure 4.**
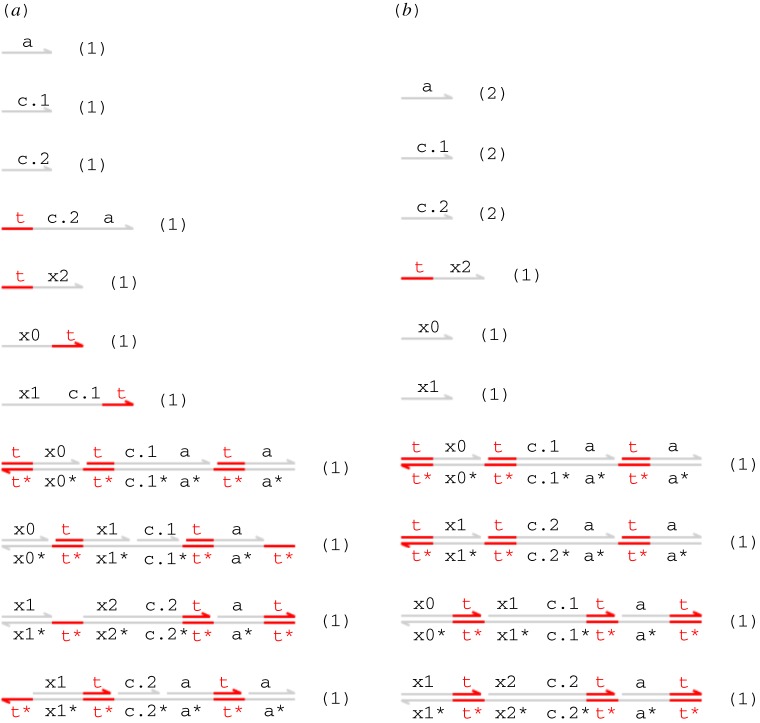
Deadlock states for the two faulty transducers in series. (*a*) State 1 (error); (*b*) state 2 (success). (Online version in colour.)

When we use prism to check these two queries, we find that the second is true but the first is false. In fact, we find that there are two deadlock states in the model, one where “all_done” is false and one where it is true. We can visualize both states using the Visual DSD tool, as shown in figure 4. State 2, on the right-hand side, represents the case where the system has executed correctly and indeed this is the result that we would anticipate: the state contains the output strand 〈t^∧^ x2〉 along with the inert garbage left over from complete execution of the two transducer gates. State 1, however, is incorrect: even though the output strand 〈t^∧^ x2〉 is produced, we see that some constituent complexes of the transducers are left in a reactive state, with exposed toehold domains.

When checking that the first query above is false, prism also produces a *counterexample* in the form of a path through the model that leads to a deadlock state where “all_done” is not true (state 1 from figure 4). Analysing this path reveals exactly *how* the system can fail. The first few reactions proceed as one would expect.


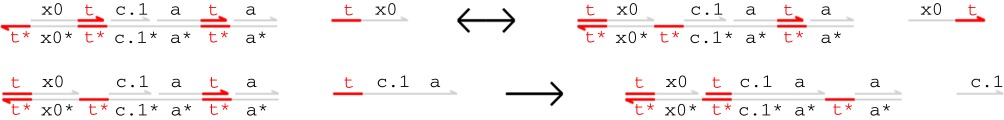


The problem arises because the 〈a t^∧^〉 strand released from the input complex of the *X*_0_ → *X*_1_ transducer can now interact with the output complex of the *X*_1_ → *X*_2_ transducer, causing the following reactions.


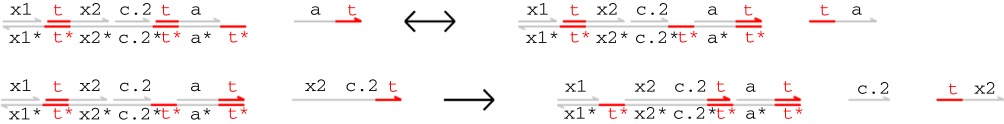


There are some subsequent reactions that tidy up as many as possible of the species with exposed toeholds. The output strand *X*_2_ is produced, as expected, but there are still some reactive species left at the end. The 〈a t^∧^〉 strand from the *X*_0_ → *X*_1_ transducer prematurely activates the *X*_1_ → *X*_2_ transducer without producing the intermediate 〈t^∧^ x1〉 signal, thereby leaving parts of the transducers unused. This happens because of *crosstalk*: the two transducers share a common recognition domain ‘a’ that allows them to interfere with each other. In contrast, the ‘new c’ declaration in the definition of the T(N,x,y) module from figure 2 enforces that the two transducers use different nucleotide sequences for their ‘c’ domains. The existence of this faulty behaviour was pointed out in Cardelli [15] and illustrated by manually tracing a path through the system. Such bugs have, however, proved to be difficult to identify manually using simulation tools. Here, we demonstrate that model checking can identify such flaws in an automatic fashion.

**Figure 5.**
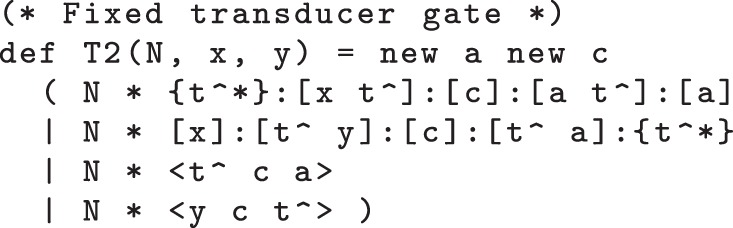
Corrected transducer gate code, with an additional ‘new a’ declaration that prevents crosstalk between different gates.

We can fix the crosstalk bug in the transducer module from figure 2 by adding an additional ‘new a’ declaration within the module definitions, as shown in the definition of the alternative T2(N,x,y) module in figure 5. This suffices to prevent crosstalk between the populations of gates that implement the different chemical reactions, because each population of gates uses different domains for their private ‘a’ and ‘c’ domains. We verify this using prism by re-running the above-mentioned queries against the same model but with occurrences of the T module replaced by T2 modules. In each case, these designs are correct: both queries are true.

### 3.2. Transducer gates: performance and reliability

Next, we examine some *quantitative* properties of the transducer gate designs from §3.1. Returning first of all to the pair of faulty transducers, we use prism to analyse the kinetics of the system. Recall that there are two possible outcomes once the system eventually terminates, one where the execution has completed successfully and one where it has not. Using the prism temporal logic queries


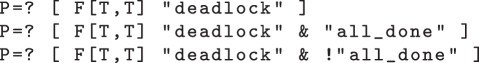


we can compute the probability that the transducer pair has, after exactly 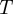 seconds: (i) terminated, (ii) terminated in error; and (iii) terminated successfully.

**Figure 6.**
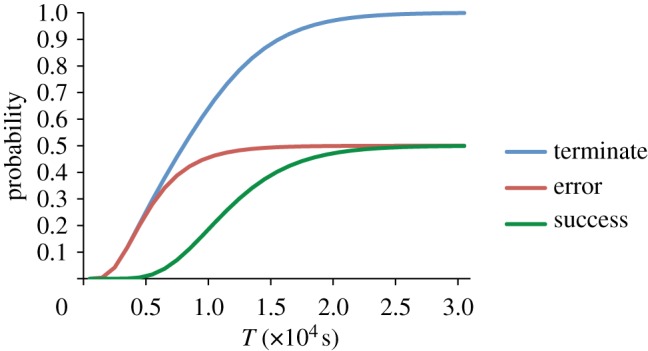
Plot showing the probability for each possible outcome of the faulty transducer pair, after *T* seconds. (Online version in colour.)


Figure 6 shows how these probabilities vary for different values of *T*. We see that the erroneous outcome is more likely to occur in the early stages than the successful outcome. This is to be expected because the error in the system arises when various intermediate reaction steps are skipped. By removing the time parameter *T* from the queries, we can use prism to compute the *eventual* probability of each outcome:

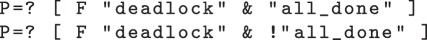


As the plot in figure 6 suggests, there is in fact an equal probability of 0.5 for each possible outcome. Intuitively, this can be explained as follows. There is a point in the execution of the system where, as described in §3.1, it is possible either for the reaction to proceed as intended or for interference between gates to occur. In fact, each of these two conflicting reactions occurs with the same rate, meaning that they are equally likely. Furthermore, each one is irreversible; so the final outcome is effectively decided at this point.

Interestingly, although a single copy of the faulty transducer pair is clearly unreliable (because it fails with probability 0.5), we can improve the overall reliability of the design by adding multiple (*N*) parallel copies of the gates. Section 4 of Cardelli [15] suggests that, if large populations of these gates execute in parallel, the additional strands available in the system should be able to ‘unblock’ the partially completed gates in the erroneous deadlock state. This hypothesis is supported by evidence from individual stochastic simulations of the system. Here, we use prism to perform a more exhaustive analysis: we compute, for different values of *N*, the expected percentage of gates in the final state of the system that are still reactive (recall from §3.1 that a reactive gate in the final state is an indicator that the transducer did not execute correctly).

The DSD code for *N* copies of the transducer pair is: S(N,x0)| T(N,x0,x1)| T(N,x1,x2). There are several different ways to compute the desired property using prism. One simple way is to use the query:


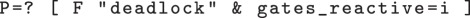


which gives the probability that there are i reactive gates in the final state of the system and, from this, compute the expected final percentage.

**Figure 7.**
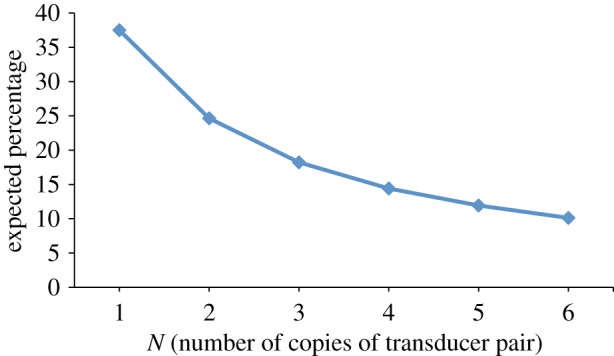
Plot showing expected percentage of leftover reactive gates in the final state of the system against the number of parallel buggy transducers—that is, the parameter N in the system S(N,x0)| T(N,x0,x1)| T(N,x1,x2). We observe that the expected number of unreactive (i.e. correctly executed) gates increases as we add more parallel copies of the buggy transducer system. (Online version in colour.)

Figure 7 plots this value for a range of values of *N*. We see that the percentage of reactive gates decreases with increasing *N*, indicating, as conjectured, that running more copies of the faulty gates in parallel (i.e. increasing *N*) means that more of the gates in the system will be used correctly.

Finally, in this section, we consider *performance* properties of DNA strand displacement systems, as opposed to the *reliability* properties discussed earlier. Seelig & Soloveichik [23] showed that the time for strand displacement circuits to execute increases linearly with the depth of the circuit. We can verify such properties computationally with our model of DNA strand displacement and prism. Using the corrected transducer design from figure 5, we constructed DSD models of the form S(1,x0)| T2(1,x0,x1)| . . .| T2(1,x { k − 1},xk), for various values of *k*, which correspond to transducer chains of increasing length. Note that we only consider a single copy of every transducer in the chain. We used the following prism temporal logic query to compute the expected time to reach a state in which all of the gates have finished executing:


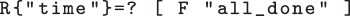


In this query, "time" denotes a simple prism reward structure (see §4.2) that assigns a reward of 1 to all states of the model. This indicates the *rate* at which reward will be accumulated, i.e. *T* units of reward for each *T* seconds spent in a state. The property above-mentioned gives the expected reward accumulated until "all_done" first becomes true, thus giving the expected execution time.

**Figure 8.**
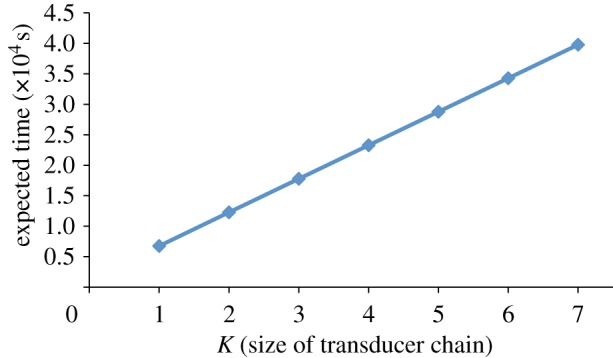
Plot showing the expected time to terminate for chains of corrected transducer gates; that is, we vary the parameter k in the system S(1,x0)| T2(1,x0,x1)| . . .| T2(1,x{ k − 1} ,xk). (Online version in colour.)

The results are shown in figure 8. We observe that there is indeed a linear relationship between the time to complete the circuit and the number of transducers in the chain, which agrees with Seelig & Soloveichik [23]. Thus, we can use probabilistic model checking to analytically investigate the kinetics of strand displacement circuits.

### 3.3. Catalyst gates

**Figure 9.**
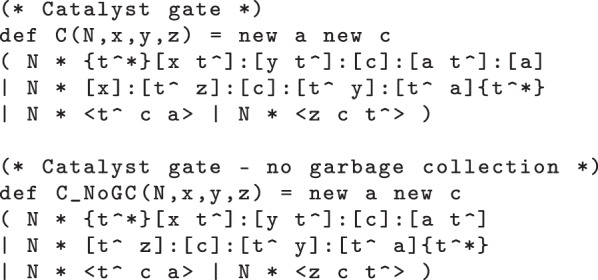
Catalyst gate code, presenting two different gate implementations: one that carries out garbage collection reactions and one that does not.

**Figure 10.**
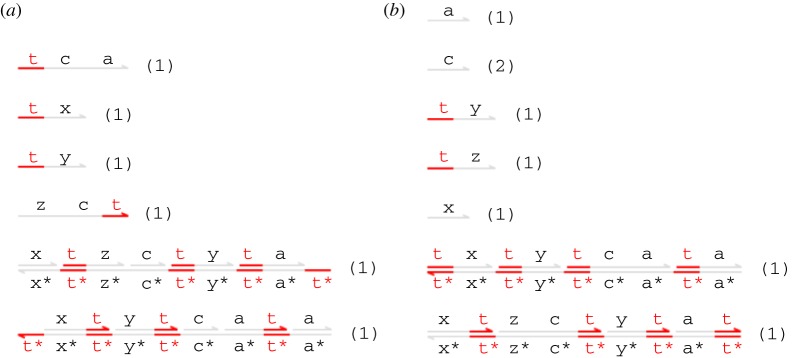
(*a*) Initial species and (*b*) expected final species for the catalyst gate C(1,x,y,z). Garbage collection results in only inert structures being present among the final species. (Online version in colour.)

We now focus on a more complicated reaction gate that models a chemical reaction *X* → *Z* catalysed by a third species *Y*, i.e. a reaction of the form *X* + *Y* → *Y* + *Z*. Figure 9 presents DSD code for a catalyst module C(N,x,y,z) that represents *N* copies of the reaction gate implementing the chemical reaction *X* + *Y* → *Y* + *Z*. This is an extension of the transducer gate, which takes advantage of the fact that the extra reactant and product are both of the same species in order to optimize the gate design. Figure 10 shows the initial and expected final states of the system for one copy of the catalyst gate, i.e. the module instantiation C(1,x,y,z). Note that the catalyst gate C(N,x,y,z) effectively implements a *catalytic reaction* *X* + *Y* → *Y* + *Z* by consuming a strand *Y* as input and producing a *different* strand *Y* as output. Even though a different strand is produced, the DNA implementation effectively emulates the function of a catalyst by producing a strand identical to the one that is consumed—hence the population of the catalyst remains constant. This is in line with the general idea of emulating chemical reactions using DNA [24]: it is not the exact species and reactions that are implemented, but equivalent ones.

In this section, we will use probabilistic model checking to investigate the relative performance of two different catalyst gate designs. In particular, we study the effect of omitting *garbage collection* from the design, i.e. the process of tidying up intermediate species into inert structures. Omitting garbage collection makes the design simpler and cheaper to implement but, as we will see, has an effect on its performance.

**Figure 11.**
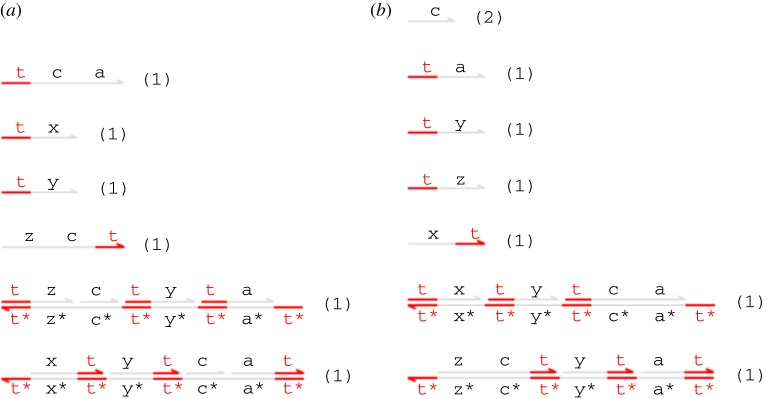
(*a*) Initial species and (*b*) expected final species for the alternative catalyst gate C_NoGC(1,x,y,z). Note that this gate does not perform the additional garbage collection reactions that produced the completely inert structures seen in figure 10. (Online version in colour.)

Figure 9 also presents DSD code for a variant catalyst module C_NoGC(N,x,y,z) that also implements *N* copies of the chemical reaction *X* + *Y* → *Y* + *Z*, but with the key difference that intermediate strands displaced from the gate during execution are *not* garbage collected by the gate structure. This means that some intermediate single-stranded fuels remain in solution after the gate has finished executing (see figure 11). We will quantify the effect that this has on the kinetics of subsequent reactions by using prism to compute the expected completion times of catalyst gates with and without garbage collection. Intuitively, we would expect that the intermediate strands that are not garbage collected will accumulate over time, gradually slowing the system down by providing a larger backward force in the kinetics.

For both variants of the catalyst gate, we adapt the earlier fragment of prism code used to identify the states of the model in which the gates have executed successfully:


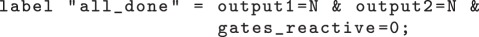


This code is as before except that, because there are now two output species we use output1 and output2 to refer to the two output signals, which are *Y* and *Z* in the case of C(1,x,y,z). We no longer require that the only reactive strands must be the output strands because this is not true for the catalyst gate without garbage collection (the leftover fuel strands are reactive). Furthermore, our definition of gates_reactive must be carefully designed to ensure a fair comparison between gates with and without garbage collection. In the case without garbage collection (C_NoGC(N,X,y,z)), the final gate structures actually contain exposed toeholds, because there are no garbage collection reactions to seal off the final toehold in the gate. Despite the fact that the final gate has an exposed toehold, the irreversible steps introduced by adding an extra domain, which was not present in the design from Cardelli [15], mean that execution of the gate is still irreversible. Because the ‘a’ and ‘c’ domains in the catalyst gate are both private to this gate, we can guarantee that no other strand in the system will be able to react with these finished gates in a productive manner. Thus, it is reasonable to adjust our definition of gates_reactive in this case so that these structures are not counted as reactive.

In the case with garbage collection (C(N,x,y,z)), the final gate structures are indeed completely sealed off. In order to make a fair comparison of the kinetics, however, we must also designate the penultimate form of each gate structure as unreactive, that is, the structure *before* the final garbage collection reaction. This is essentially saying that, for the purposes of computing the time to termination, we do not care whether the garbage collection reactions have actually taken place when we decide if the system has terminated. Without this, it would not be possible to make a fair comparison.

We first check that both designs satisfy the correctness property given earlier for transducers. Having established this, we then look at the performance of the designs, i.e. how quickly they execute. To quantify the effect of garbage collection on the kinetics of the system, we compared the behaviour of the systems S(N,x)| S(N,y)| C(N,x,y,z) and S(N,x)| S(N,y)| C_NoGC(N,x,y,z) for different values of *N*. We vary this parameter because one would expect that the negative effects of garbage collection will only begin to accumulate after a number of identical gates have been executed. Re-using the prism temporal logic query from §3.2, we compute the expected time until termination.

**Figure 12.**
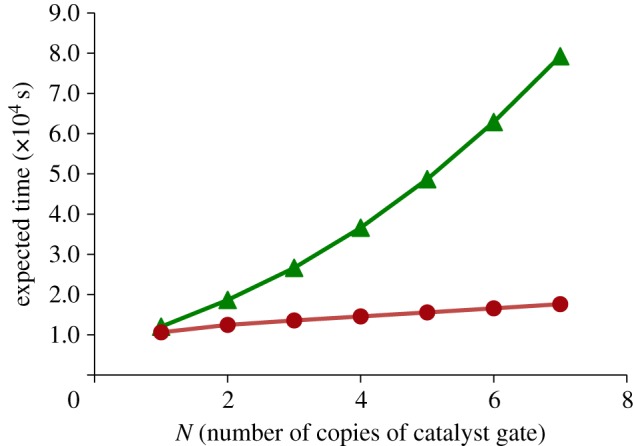
Plot of expected time to completion for *N* parallel copies of catalyst gates with (solid line with filled circles) and without (solid line with filled triangle) garbage collection. (Online version in colour.)

The results of this analysis are presented in figure 12. We observe that, as *N* increases, the expected completion time for the gates without garbage collection increases faster than for the gates with garbage collection. This confirms our intuition that accumulating waste strands from earlier executions of the gate exerts an additional backward force on subsequent executions of the gate, gradually slowing the system down.

### 3.4. Approximate majority

We now use the catalyst gates from §3.3 to implement a larger system—the approximate majority population protocol of Angluin *et al.* [25]—using DNA strand displacement. The following chemical reactions implement the approximate majority population protocol:
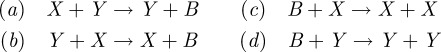


When an *X* and a *Y* meet, reactions (*a*) and (*b*) convert one of them into an auxiliary species *B* with equal probability. Then, when a *B* meets an *X* or *Y*, reactions (*c*) and (*d*) convert the *B* to match the species that it encountered. In this second step, the probability of a *B* encountering an *X* as opposed to a *Y* depends on the initial populations of *X* and *Y* in the system, and this fact allows the system to amplify any excess population of one species over the other to converge on a consensus in which all of the species are converted either to *X* or to *Y*. Furthermore, it was proved by Angluin *et al.* [25] that the system converges with high probability to the population that was initially in the majority, if the original margin is sufficiently large.

**Figure 13.**
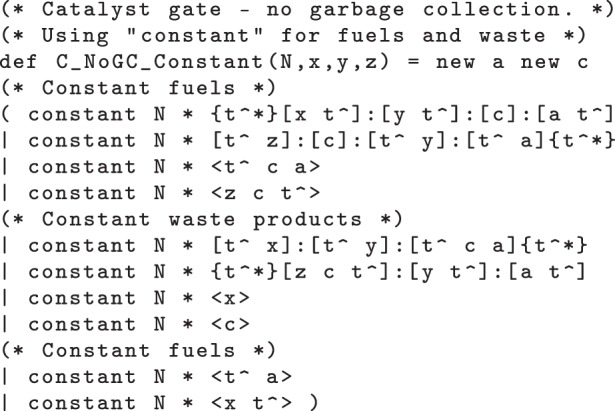
DNA strand displacement (DSD) code for a catalyst gate, which extends the C_NoGC gate from figure 9 by using the constant keyword from the DSD language to abstract away from population changes due to accumulation of waste and depletion of fuel.

Note that the above-mentioned reactions all involve catalysts, like those from §3.3. Thus, we can use the catalyst gates discussed therein to implement this system of chemical reactions in the DSD language, as shown in the code in figure 13. In order to reduce the number of reactions and to make model checking more tractable, we will use a catalyst gate without garbage collection. Even with this simplification, however, the fact that there are cycles in the chemical reactions means that the system can potentially use all of the available fuel, which causes the state space to grow enormously. To counteract this effect, we modify the gate designs from §3.3 further, using the constant keyword of the DSD language. This keyword declares that the population of a particular species should be held constant across all reactions in the system, even those reactions where it is produced or consumed. This approximation can be used when the species is in excess, allowing us to abstract away from depletion of fuels and accumulation of waste, in cases when these species are in very high concentrations. This helps us to greatly restrict the size of the state space in the prism model, by essentially collapsing any states that differ only by their populations of fuels and/or waste products.

Because the population protocol is not *guaranteed* to form a consensus around the species that was initially in the majority, we use prism to compute the probabilities of reaching each consensus state (*X* or *Y*) in the DNA implementation, given different initial populations of the species. We constructed prism models for various input populations and computed the probabilities of ending up with the two possible consensus values. Care must be taken because, even when a consensus has been achieved, the resulting signal strands can still speculatively bind to the remaining fuel gates, even though the gate will never execute fully (because we have reached a consensus, there are no different input species to bind and complete the reaction process) and the strand must therefore eventually unbind again. We use prism variables output_x and output_y, which return the number of individuals in the two consensus states, taking these transient structures into account in the definitions. The required queries in prism are then:


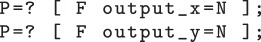


**Figure 14.**
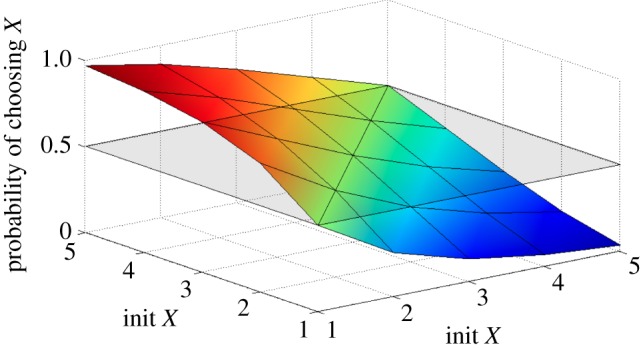
Surface plot which shows the probability of reaching a consensus of *X*, for various initial populations of *X* and *Y*. (Online version in colour.)

As a sanity check, we used the following query:


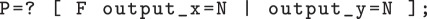


to check that the probability of eventually ending up in *either* consensus state is 1 in all cases. Thus, it suffices to study just one of the two outcomes. Figure 14 shows a plot of the probability of finishing in consensus state *X* for initial populations of *X* and *Y* ranging between 1 and 5. Table 2 shows the same values. If *X* or *Y* equals zero, then no reactions can take place, and for total initial populations exceeding 10, the model becomes too large to handle. We observe that, if the initial populations of *X* and *Y* are the same, then the system is equally likely to form a consensus around either *X* or *Y*. This is illustrated by the contour line at the 0.5 level in figure 14. However, as the initial excess of one species increases, the probability of ending up in that consensus state increases rapidly, so that when all but one of the initial species are *X* (say), then the probability of forming a consensus around *X* is almost one, as we would expect.

**Table 2.** Probability of reaching a consensus of *X*, for various initial populations of *X* and *Y*.

	*Y* = 1	*Y* = 2	*Y* = 3	*Y* = 4	*Y* = 5
*X* = 1	0.5000	0.2531	0.1290	0.0658	0.0334
*X* = 2	0.7468	0.5000	0.3156	0.1917	0.1131
*X* = 3	0.8709	0.6843	0.5000	0.3462	0.2299
*X* = 4	0.9341	0.8082	0.6537	0.5000	0.3651
*X* = 5	0.9665	0.8869	0.7699	0.6349	0.5000

**Figure 15.**
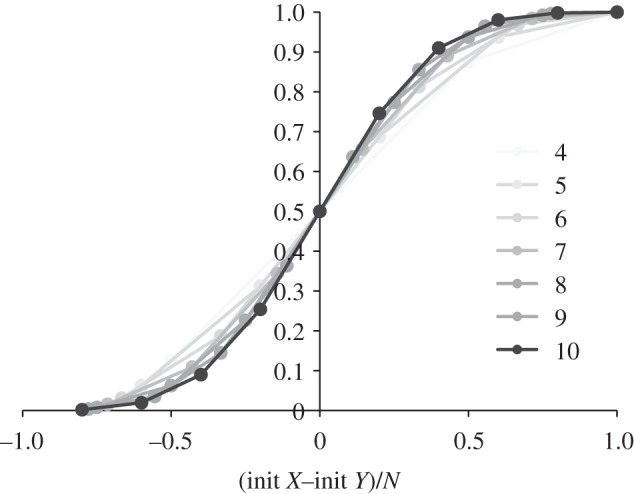
Probability of reaching a consensus of *X*, plotted against (*X*_0_ − *Y*_0_ )/*N*, which is the difference between the initial populations of *X* and *Y*, relative to the total initial population *N* = *X*_0_ + *Y*_0_ . Results are shown for *N* = 4 … 10.

Theoretical results from Angluin *et al.*  [25] show that the correct consensus is achieved with high probability if the *margin* between the initial numbers of *X* and *Y* is above 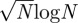, for large total initial population *N*. So, in figure 15, we also plot the probability of reaching a consensus of *X* against (*X*_0_ − *Y*_0_ )/*N*, which is the initial difference between *X* and *Y* relative to the total initial population *N* = *X*_0_ + *Y*_0_ , for *N* varying between 4 and 10. Here, we see more clearly how the probability grows with the increase in the size of the margin |*X*_0_ − *Y*_0_|/*N*. Figure 15 also illustrates how, for larger population sizes, we see an increasingly clear threshold above which consensus is achieved with high probability.

Finally, it is worth remarking that an analysis of this system in terms of ordinary differential equations (ODEs) has a number of limitations. In particular, the simulation of the ODE model gives us the *average behaviour* of the system, but not the *probability* of computing the majority. Specifically, because the system is inherently stochastic, there is always a chance that the minority wins, whereas in the ODE model the majority always wins. Furthermore, in cases where both species have equal initial populations the ODE model never converges to a majority, while the stochastic model does converge. Thus, to correctly analyse the behaviour of the system, a detailed analysis of the chemical master equation is required, which is analytically unsolvable in this case. Another alternative is to simply run large numbers of stochastic simulations, though this is also intractable, owing to the very low probability of error as the number of molecules increases.

Thus, probabilistic model checking allows us to analyse properties that would be considerably more difficult to obtain using other techniques. Essentially, probabilistic model checking can be viewed as an automated way to solve the chemical master equation for small but non-trivial model sizes. In particular, it allows us to determine the *distribution* of the system, i.e. the individual possible outcomes of the system and their corresponding probabilities.

## 4. Methods

### 4.1. Model simulation in DNA strand displacement

The syntax of DSD described in §2 interprets systems as well-mixed solutions: hence we impose a standard set of structural equivalence rules (see Lakin & Phillips [2] for more details). In addition, we assume that no long domain and its complement are simultaneously unbound anywhere in the system. This *well-formedness* restriction ensures that two species can only interact with each other via complementary toeholds.

**Figure 16.**
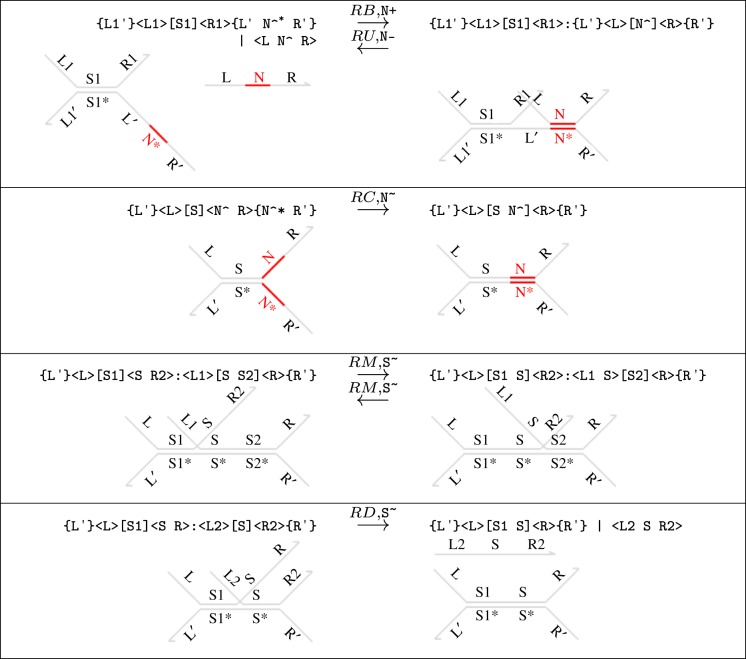
Elementary reduction rules of the DSD programming language. We let S^∼^ denote the migration rate of a domain sequence S and fst(S) denote its first domain. We also let N+ and N − denote the binding and unbinding rates, respectively, of a toehold N^∧^. We assume that fst(R2) ≠ fst(S2) for rule (RM). This ensures that branch migration is maximal along a given sequence and that rules (RM) and (RD) are mutually exclusive. (Online version in colour.)


The rules in figure 16 present reduction rules that formalize DNA interactions in the DSD language. The arrows are labelled with rates that are used to parametrize an exponential rate distribution. Rules (RB) and (RU) define reversible toehold-mediated binding and unbinding reactions between a single strand and a double-stranded gate complex. Rule (RC) allows complementary toeholds to hybridize if they are present in opposite overhanging strands of a gate. This situation could arise when an incoming strand contains multiple toeholds that match up to exposed complementary toeholds in the gate. We do not provide versions of rules (RB), (RU) and (RC), where the complementary domain is not a toehold because our well-formedness assumption ensures that the only complementary domains exposed simultaneously are toeholds. Rules (RM) and (RD) present primitive branch migration and strand displacement reactions, respectively. Rule (RD) can be thought of as the special case of (RM) where there are sufficiently many matching domains for the branch migration to make it right to the end of the strand. The additional assumption that fst(R2) ≠ fst(S2) in rule (RM) ensures that we only derive maximal branch migration reactions and also ensures that rules (RM) and (RD) are mutually exclusive. As an example, the following steps illustrate the application of the sequence of rules (RB), (RD) and (RC) on an initial system 〈t^∧^ x u^∧^〉| {t^∧^*}[x]{u^∧^*}

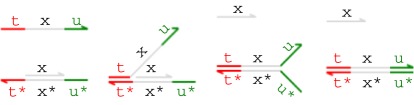


The rules of figure 16 define the fundamental DNA reactions that we consider in this paper. We do not consider reactions that would form chain polymers or complex secondary structures, or leak reactions. To complete the definition of reduction, however, we must also provide additional contextual rules that allow these primitive steps to occur in richer contexts. For example, rule (RB) allows a strand to bind to the bottom-right overhanging strand of a segment, whereas in reality it could bind to any of the overhangs (provided that there is a complementary toehold available). We also require that these reactions can take place partway along more complex molecules. Thus, we require each reduction rule to be closed under rotation and mirroring of individual DNA species about the axis of the double-stranded backbone, and under concatenation of additional segments onto either end of the gate complex involved in a particular reaction. We also lift the rules to transform systems involving parallel compositions and name restrictions in the standard way. We refer the reader to Lakin & Phillips [2] for further details.

The reduction rules in figure 16 provide a detailed model of DNA strand displacement reactions between single strands and gate complexes. In fact, for our purposes a simpler model would suffice. Hence, we use a *merged* reduction semantics for the DSD language, in which we model toehold binding reactions as having a finite rate and all other reactions as instantaneous. In particular, we consider gates to be equivalent up to branch migration; so if two gates differ only by applications of rule (RM), we treat them as if they were the same gate. These simplifications are based on the assumption that toehold binding steps are sufficiently slow to be rate limiting, which is valid in the limit of low concentration.

We write 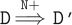 to denote a merged reduction from 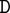 to 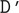. Formally, 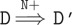 means that 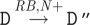 and 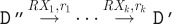 both hold, for some 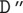, and where none of the (RX_*i*_) rules are repeat occurrences of (RB). Furthermore, because we are assuming that branch migration is included in the structural equivalence relation, there should be no occurrences of (RM) either. In order to improve efficiency and reduce the size of the resulting model, we ignore *unproductive* reactions where a strand binds onto a gate but cannot perform any subsequent reaction other than an unbinding. This corresponds to merged reductions of the form 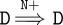.

The merged reduction relation defined earlier is the *Infinite* semantics from Lakin & Phillips [2]. This is the most abstract model of DNA strand displacement interactions defined in that paper, and allows us to produce more compact models for verification. In this paper, we phrase all models in terms of these merged rules, which allow us to translate a collection of DNA molecules into a system of chemical reactions for analysis. Under this condensed semantics, the four reactions from figure 1 are combined into the following single reaction.


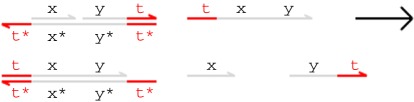


Where possible (i.e. where models do not become too large for verification), we have also re-run the experiments in this paper using models obtained with DSD's *Default* semantics, observing an overall slow down in reaction time, but otherwise identical patterns in behaviour. For all simulations and analysis, the kinetic rates of toehold binding, toehold unbinding and branch migration were based on the experimental measurements of Zhang & Winfree [26]. These rates were in turn used to derive the corresponding simulation and analysis times, in seconds.

### 4.2. Probabilistic model checking in prism

prism [7] is a probabilistic model checking tool developed at the Universities of Birmingham and Oxford. It provides support for several types of probabilistic models, including CTMCs, which we use here. Models are specified in a simple, state-based language based on guarded commands. Support for several other high-level model description languages has also been made available through language-level translations to the prism modelling language. For example, prism has the ability to import SBML [27] specifications, which have an underlying CTMC semantics. Translations from stochastic process algebra such as PEPA and the stochastic calculus [28] have also been developed.

Formally, letting R_≥0_ denote the set of non-negative reals and *AP* denote a fixed, finite set of atomic propositions used to label states with properties of interest, a CTMC is a tuple (*S*,**R**,*L*) where:

— *S* is a finite set of *states*;
— **R**:(*S* × *S*) → R_≥0_ is a *transition rate matrix*;
— *L*:*S* → 2^*AP*^ is a *labelling* function which associates each state with a set of atomic propositions.

The transition rate matrix **R** assigns rates to each pair of states, which are used as parameters of the exponential distribution. A transition can only occur between states *s* and *s*′ if **R**(*s*,*s*′)> 0 and, in this case, the probability of the transition being triggered within *t* time units is 1 − e^− **R**(*s*,*s*′)*t*^. Typically, in a state *s*, there is more than one state *s*′ for which **R**(*s*,*s*′)> 0; this is known as a *race condition* and the first transition to be triggered determines the next state. The time spent in state *s* before any such transition occurs is exponentially distributed with the rate 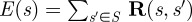, called the *exit rate*. The probability of moving to state *s*′ is given by **R**(*s*,*s*′)/*E*(*s*).

A CTMC can be augmented with *rewards*, attached to states and/or transitions of the model. Formally, a *reward structure* for a CTMC is a pair (*c*,*C*) where

— *c*:*S* → R_≥0_ is a *state reward function*;
— *C*:(*S* × *S*) → R_≥0_ is a *transition reward function*.

State rewards can represent either a quantitative measure of interest at a particular time instant (e.g. the number of phosphorylated proteins in the system) or the rate at which some measure accumulates over time (e.g. energy dissipation). Transition rewards are accumulated each time a transition occurs and can be used to compute, e.g. the number of protein bindings over a particular time period.

prism can then be used to specify and verify a range of properties of CTMCs, including those expressed in the logic CSL and the reward-based extension of Kwiatkowska *et al.*  [20]. The underlying computation performed to apply probabilistic model checking involves a combination of graph–theoretical algorithms (e.g. to construct and explore models) and numerical computation methods (e.g. to calculate probabilities or reward values). For the latter, prism typically solves linear equation systems or performs transient analysis. Owing to the size of the models that need to be handled, it uses iterative methods rather than direct methods. For solutions of linear equation systems, it supports a range of well-known techniques, including the Jacobi, Gauss-Seidel and SOR (successive over-relaxation) methods; for transient analysis of CTMCs, it employs uniformization.

The prism tool also offers a graphical user interface with a range of functionality. First, it facilitates construction and editing of prism models and property specifications. In addition, it includes a graph-plotting component for visualization of numerical results from probabilistic model checking. It also provides a tool to perform manual execution and debugging of probabilistic models, based on an underlying discrete-event simulation engine. Another use of this engine is to generate approximate solutions for the numerical computations that underlie the model checking process, by applying Monte Carlo methods and sampling. These techniques offer increased scalability, at the expense of numerical accuracy. The main strength of prism, though, and the probabilistic model checking techniques that it implements, is the ability to compute quantitative properties exactly, based on exhaustive model exploration and numerical solution.

## 5. Discussion

We have introduced a modified version of Cardelli's two-domain gate designs [22], which is amenable to verification yet retains the key simplification of the two-domain design: initial species contain no overhangs and can thus be constructed by inserting breaks into one strand of a simple double-stranded DNA complex. This should give higher yields compared with simple annealing of single strands in a test tube.

We have demonstrated that probabilistic (and non-probabilistic) model checking can be used to verify a wide range of properties of individual circuit components constructing using this design. We showed that the prism model checker can detect bugs owing to crosstalk between gates, analyse quantitative properties such as reliability and performance, and compute the probability of different possible outcomes of the gates' execution. These techniques can be particularly useful during the initial stages of gate design. Even model checking a single gate executing in isolation, as in §3.2, can help us to identify errors in the design that would be difficult to quantify using simulation-based methods. Although multiple simulation runs can be used to approximate the probability of a given error, performing large numbers of simulations can be time-consuming, particularly in cases such as figure 7, where the error probability is low for large number of gates. More importantly, model checking can be used to identify the source of an error, by providing a specific execution trace of the behaviour that leads to its occurrence. As illustrated in §3.3, we can also use model checking to investigate the potential of different system designs, even when analysed using relatively small numbers of inputs. Finally, we have shown how applying additional abstractions to the populations of fuel and waste species can allow us to scale up to verifying more complicated systems, such as the approximate majority population protocol [25].

Nonetheless, model checking has its limitations. As the species populations grow, the number of reaction interleavings explodes, which causes problems for naively scaling up to larger systems. Table 3 shows statistics for a selection of the largest models that we used to generate the results in §3 (model checking was run on a 2.80 GHz Dell PowerEdge R410 with 32 GB of RAM). The table shows the size of each model and the time required to check a single property. As expected, model sizes grow rapidly as population sizes are increased, meaning that models larger than those shown in the table could not be analysed. In §3.4, we had to approximate the populations of fuel and waste species in the model as constant in order to prevent the state space from becoming too large to generate. This effect can be mitigated to an extent, for example, by careful gate design: our modified two-domain gates were deliberately constructed to minimize the number of asynchronous steps required for garbage collection and sealing off used gates, which greatly expand the state space. We also used a high level of abstraction (the *Infinite* semantics of DSD [2]) to reduce the number of reactions in the model as far as possible. However, even with the cleverest gate design, the sheer number of interleavings will eventually become too great.

**Table 3.** Model checking statistics: model sizes and run-times.

example	parameters	states	time (s)
buggy transducers (§3.2)	*N* = 4	57 188	4
	*N* = 5	284 641	26
	*N* = 6	1 160 292	145
approx. majority (§3.4)	*X* = 3,*Y* = 5	240 286	266
	*X* = 4,*Y* = 5	674 066	860
	*X* = 5,*Y* = 5	1 785 250	2602

One key challenge is to extrapolate the results from model checking relatively small systems to systems with higher numbers of molecules. Being able to identify design flaws in individual system components, such as the buggy transducer gate in §3.1, is already valuable because the flaws are still likely to occur when the component is present in larger numbers or is part of a more complex design. On the other hand, our full-system model checking does not verify interactions with an arbitrary environment. For example, the buggy transducer gate would appear to work correctly when model-checked in isolation—the unwanted crosstalk only becomes apparent when two gates are model-checked together. To help address this, we can selectively model check a given gate design with all of the remaining gates in the system, in order to identify possible interferences.

For quantitative properties, such as performance or reliability, we showed in §3.2 that it is already possible to make comparisons between gate designs using relatively small numbers of molecules. Furthermore, for certain categories of circuits, such as those involving localized strands tethered to the surface of DNA origami [29], the internal behaviour of each origami circuit can be analysed independently, and then used to accurately predict the behaviour of potentially millions of circuits in solution. This is because localization significantly reduces cross-talk between circuits, allowing them to be accurately analysed in modular and scalable ways. We also note that, in the context of cell signalling pathways, prism has been used successfully to evaluate regulation mechanisms for the fibroblast growth factor pathway [6]: behavioural predictions from a prism model over small population numbers were later validated experimentally [30]. In this paper, we were able to reproduce behavioural trends previously analysed in a theoretical setting [23,25]. In the future, we plan to investigate experimental validation of our analysis techniques for DNA strand displacement circuit designs.

Other important areas for research include developing techniques to further improve the scalability of probabilistic model checking on DNA designs—for example, through the construction of *abstractions* or by analysing systems in a *compositional* manner. Promising directions for the former include sliding window abstractions [31], which optimize the analysis of temporal system properties by restricting analysis to a particular subset of the state space for each phase of its evolution, and the stochastic hybrid model of [32] for analysing systems in which some populations are present in small numbers and others in large numbers. Abstractions will also become essential when modelling Turing-powerful computation with DNA strand displacement, because the corresponding reaction network is of potentially unbounded size. In this case, a notion of dynamically generated reactions is needed, as discussed in Lakin & Phillips [2].

Regarding compositional techniques, it may be beneficial to consider stochastic Petri nets [33], which are an alternative means of representing the behaviour of strand displacement systems, and have already been applied to systems and synthetic biology [34]. In this approach, places correspond to DNA species and transitions to the chemical reactions between them. Previous work has explored compositional model checking of (non-stochastic) *modular* Petri nets [35] composed by transition sharing. In fact, in the context of Petri net-based models for strand displacement systems, it would be advantageous to consider compositions based on both sharing of transitions and of places.

We believe that advances in abstraction techniques and compositional model checking will be vital in order to apply model checking to larger DNA strand displacement systems. In fact, the two-domain scheme is an excellent framework for research into compositional verification of strand displacement circuits because the restricted syntax makes it straightforward to compute, for any gate complex, the set of all single-stranded species that could interact with the complex. This is a much more challenging problem for more general strand displacement schemes where strands can contain arbitrary domains. This insight could allow us to relate every gate complex with a finite-state automaton describing its possible interactions with the environment, which could form the basis for a compositional verification technique. Just as we used generic temporal logic formulae to characterize correct final states of the reaction gates examined in §3, for compositional model checking we would need to identify temporal logic formulae that characterize valid sequences of interactions between the gate and its environment. We would hope to prove that, if a particular set of gates all satisfy those formulae, then any cascade comprising just those gates would be correct in some sense. Such techniques will become increasingly important as larger and more complex strand displacement systems are constructed, such as Qian & Winfree's  [14] four-bit square root circuit.

## Footnotes

1 http://lepton.research.microsoft.com/webdna
